# Altered amygdala-based functional connectivity in individuals with attenuated psychosis syndrome and first-episode schizophrenia

**DOI:** 10.1038/s41598-020-74771-w

**Published:** 2020-10-19

**Authors:** Woo-Sung Kim, Guangfan Shen, Congcong Liu, Nam-In Kang, Keon-Hak Lee, Jing Sui, Young-Chul Chung

**Affiliations:** 1 Department of Psychiatry, Medical School, Jeonbuk National University, Geonjiro 20, Jeonju, Korea; 2 Department of Psychiatry, Jeonbuk National University Hospital, Jeonju, Korea; 3 Research Institute of Clinical Medicine of Jeonbuk National University-Biomedical Research Institute of Jeonbuk National University Hospital, Jeonju, Korea; 4 Department of Psychiatry, Maeumsarang Hospital, Wanju, Jeollabuk-do Korea; 5 Brainnetome Center and National Laboratory of Pattern Recognition, Institute of Automation, Chinese Academy of Sciences, Beijing, 100190 China; 6 University of Chinese Academy of Sciences, CAS Center for Excellence in Brain Science and Intelligence Technology, Chinese Academy of Sciences, Beijing, 100049 China

**Keywords:** Neuroscience, Medical research

## Abstract

Altered resting-state functional connectivity (FC) of the amygdala (AMY) has been demonstrated to be implicated in schizophrenia (SZ) and attenuated psychosis syndrome (APS). Specifically, no prior work has investigated FC in individuals with APS using subregions of the AMY as seed regions of interest. The present study examined AMY subregion-based FC in individuals with APS and first-episode schizophrenia (FES) and healthy controls (HCs). The resting state FC maps of the three AMY subregions were computed and compared across the three groups. Correlation analysis was also performed to examine the relationship between the Z-values of regions showing significant group differences and symptom rating scores. Individuals with APS showed hyperconnectivity between the right centromedial AMY (CMA) and left frontal pole cortex (FPC) and between the laterobasal AMY and brain stem and right inferior lateral occipital cortex compared to HCs. Patients with FES showed hyperconnectivity between the right superficial AMY and left occipital pole cortex and between the left CMA and left thalamus compared to the APS and HCs respectively. A negative relationship was observed between the connectivity strength of the CMA with the FPC and negative-others score of the Brief Core Schema Scales in the APS group. We observed different altered FC with subregions of the AMY in individuals with APS and FES compared to HCs. These results shed light on the pathogenetic mechanisms underpinning the development of APS and SZ.

## Introduction

Schizophrenia (SZ) is a severe neuropsychiatric disorder. A leading hypothesis is that SZ is a brain disconnection syndrome involving abnormal interactions among widespread brain networks^1,2^. Widespread network disconnectivity in SZ has been reported using both seed-based analysis and independent component analysis (ICA) with rs-fMRI data (for a review, see Ref.^3^). More specifically, most seed-based studies have found decreased connectivity^4–7^, whereas ICA studies have reported both increases^8,9^ and decreases^10,11^. The medial frontal cortex (mPFC) has often been shown by both approaches to be involved in aberrant connectivity in SZ^3^. The main findings of rs-fMRI studies in first-episode schizophrenia (FES) are similar. Robust regional brain changes were found in the prefrontal and temporal lobes, including decreased functional connectivity (FC) in the mPFC^12,13^, dorsal lateral prefrontal cortex (DLPFC)^14^, orbital frontal cortex^13^, and ventrolateral prefrontal cortex^13,14^ and increased or decreased FC in the temporal lobe, with prominence in the left superior temporal gyrus (STG)^15,16^ (for a review, see Ref.^17^).

Individuals with attenuated psychosis syndrome (APS), the most common type of clinical high risk (CHR) state for psychosis, provide an opportunity to explore the earliest pathophysiology of SZ given the consistent transition risk of 22% after 1 year and 36% after 3 years^18^. Studies on the resting-state FC (rsFC) in subjects with APS is ongoing, and evidence is limited. Most previous FC studies have investigated networks seeded from a priori defined regions of interest (ROIs): the amygdala (AMY)^19^, cerebellum^20^, posterior cingulate cortex (PCC)^21^, striatum (ST)^22,23^, STG^24^, and thalamus^25^. Overall, the data points to reduced FC in the corticolimbic and corticostriatal circuits and mixed findings pertaining to FC in the corticothalamic circuits.

The AMY is composed of structurally and functionally distinct nuclei that contribute to the processing of emotion through interactions with other subcortical and cortical structures. The laterobasal AMY (LBA) facilitates associative learning processes such as fear conditioning through afferent nerves from cortical and subcortical regions, including the thalamus, hippocampus, and prefrontal cortex^26,27^. The centromedial AMY (CMA) plays an important role in generating behavioral responses through projections to the brainstem, as well as cortical and striatal regions^26,28^, and facilitates attention to salient stimuli^29^. The superficial AMY (SA) lies adjacent to the laterobasal group and includes the cortical nuclei involved in olfactory^30,31^ and affective processes^32^. Despite the potential importance of understanding AMY-related dysfunction in APS and SZ, relatively few studies have directly examined alterations in AMY connectivity across the illness phases; only five studies on SZ^19,33–36^ and two studies on APS^19,37^ have done so. Of note, only two studies on SZ used subregions of the AMY as a seed ROI^34,36^. Given the different roles of AMY subregions and the aberrant rsFC of specific AMY subregions in SZ, it is crucial to investigate rsFC in the AMY subregions of individuals with APS. To our knowledge, no prior work has investigated FC in individuals with APS using subregions of the AMY as a seed ROI.

We hypothesized that participants with APS would exhibit altered FC between subregions of the AMY and other brain regions compared to healthy controls (HCs) and patients with SZ. The present study examined AMY subregion-based connectivity in individuals with APS, patients with FES, and HCs. The rsFC maps of the three AMY subregions were computed and compared across the three groups. Given the role of AMY in the formation of self-referential schemas^38^ and the close association between negative schemas and positive symptoms^39^, exploratory analyses were performed to examine the correlations between the Z-values of regions showing significant group differences and Positive and Negative Syndrome Scale (PANSS; Refs.^40,41^ and Brief Core Schema Scales (BCSS; Ref.^42^) scores.

## Results

### Demographic and clinical characteristics

The FES group comprised nine patients with SZ and 16 patients with schizophreniform disorder. All individuals with APS were classified as the attenuated psychosis group with subthreshold intensity. There were significant differences in sex, education, and BCSS score among the three groups. Post hoc results showed that education level was significantly lower in the APS and FES groups compared to the HC or FES group. On the BCSS, subscale scores of the APS and FES groups were significantly lower than those of the HC group. On the PANSS, total and subscale scores were all significantly lower in the APS group compared to the FES group (Table 1).

**Table 1:** Demographic and clinical characteristics of patients with APS, FES and HC.

Characteristics	APS (n = 22)	FES (n = 25)	HC (n = 31)	*p*-value^a^
**Age (years)**	22.41(5.16)	24.48(3.45)	24.35(2.35)	0.102
**Sex**				
Male (n)	19	9	14	0.001^b^
Female (n)	3	16	17	
**Education (years)**	12.09 (1.60)***^,†††^	13.92 (1.93)^††^	15.29 (1.07)	< 0.001
**Duration of illness (months)**	24.64 (23.60)***	6.70 (7.19)	–	0.001^c^
**SOPS**				
Positive symptoms	9.00 (9.60)	–	–	–
Negative symptoms	9.00 (7.26)	–	–	–
Disorganization symptoms	1.73 (1.93)	–	–	–
General symptoms	5.64 (3.08)	–	–	–
Total score	25.36 (16.72)	–	–	–
**PANSS**				
Positive symptoms	9.95 (4.20)***	17.37 (7.10)	–	< 0.001^c^
Negative symptoms	9.73 (4.79)*	14.18 (5.94)	–	0.012^c^
General psychopathology	23.68 (9.02)**	32.59 (9.07)	–	0.001^c^
Total symptoms	43.36 (16.42)***	64.15 (15.81)	–	< 0.001^c^
**BCSS**				
Negative self	12.19 (5.87)^†††^	10.40 (7.02)^††^	3.81(8.26)	< 0.001
Negative others	9.79 (6.32)^†††^	9.80 (7.00)^†††^	2.32(3.11)	< 0.001
Positive self	7.82 (6.37)^†††^	8.68 (5.49)^†††^	15.34(3.60)	< 0.001
Positive others	6.45 (5.27)^†^	7.28 (5.61)	10.52(5.19)	0.015
**Medication**				
Naive/free	15/7	8/8	–	–
chlorpromazine equivalent (mg)	–	348.89 (218.67) (n = 9)	–	–

Data given as mean (SD).

*APS* attenuated psychosis syndrome, *BCSS* brief core schema scale, *FES* first episode schizophrenia, *HC* healthy control, *PANSS* positive and negative syndrome scale.

^a^Significant *F* statistic for the one way between group ANOVA.

^b^Significant F statistic for the Chi-square test.

^c^Significant *T* statistic for the two sample *t*-test.

*P < 0.05, **P < 0.01, ***P < 0.001 compared to FES.

^†^*P* < 0.05, ^††^*P* < 0.01, ^†††^*P* < 0.001 compared to HC.

### AMY seed-based functional connectivity

**Figure 1:**
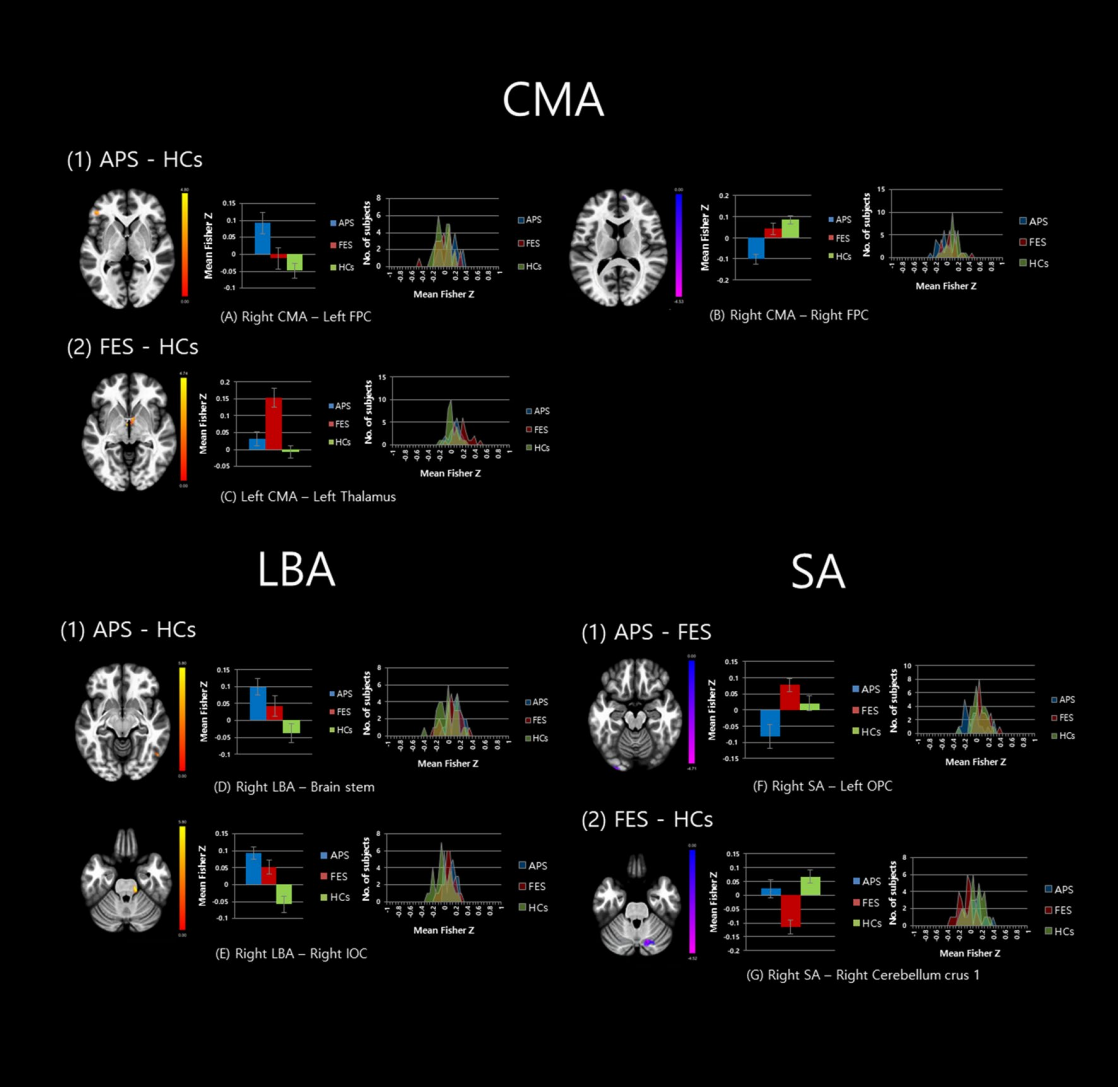
Altered amygdala subregion-based functional connectivity among the attenuated psychosis syndrome, first episode schizophrenia and healthy control groups. Significant differences were revealed between the (**A**) Right centromedial amygdala (CMA) and Left frontal pole cortex (FPC); (**B**) Right CMA and Right FPC; (**C**) Left CMA and left thalamus; (**D**) Right laterobasal amygdala (LBA) and Brain stem; (**E**) Right LBA and Right inferior occipital cortex; (**F**) Right superficial amygdala (SA) and Left occipital pole cortex (OPC); (**G**) Right SA and Right cerebellum crus 1. The functional connectivity Z values of regions showing significant differences are presented in bar graph.

In the post hoc pairwise comparisons, the APS group showed significantly increased FC between the right CMA and left frontal pole cortex (FPC) (*t* = 4.84, *p* < 0.05) and between the right LBA and brain stem (*t* = 5.67, *p* < 0.05) as well as the right inferior lateral occipital cortex (LOC) compared to HCs. The APS group also exhibited significantly decreased FC between the right CMA and right FPC (*t* = 5.32, *p* < 0.05). On the other hand, the APS group exhibited decreased FC between the right SA and the left occipital pole cortex (OPC) (*t* = 5.53, p < 0.05) compared to the FES group. The FES group exhibited significantly increased FC between the left CMA and left thalamus (*t* = 5.98, p < 0.05) and decreased FC between the right SA and right cerebellum crus 1 (*t* = 4.93, p < 0.01) (Table 2 and Fig. 1).

**Table 2:** Post-hoc analysis results of seed to voxel functional connectivity among APS (n = 22), FEP (n = 25), and HC (n = 31).

Seed region	MNI coordinate	Cluster size	*t* value	Effect size	*p*-FWE	*p*-FDR	*p*-unc	Name (voxel size-region)
**APS > HC**								
Right centromedial amygdala	− 46 38 − 2	97	4.84	0.24	0.040	0.031	0.001	60—left frontal pole cortex
Right laterobasal amygdala	12 − 26 − 26	82	5.67	0.28	0.087	0.049	0.003	65—brain stem
	52 − 64 − 10	78	5.75	0.23	0.104	0.049	0.003	41—right inferior lateral occipital cortex
**APS < HC**								
Right centromedial amygdala	0 64 4	91	5.32	0.24	0.053	0.031	0.002	66—right frontal pole cortex
**FES > APS**								
Right superficial amygdala	− 34 − 94 − 12	98	5.53	0.23	0.044	0.048	0.001	63—left occipital pole cortex
**FES > HC**								
Left centromedial amygdala	− 4 − 6 − 4	97	5.98	0.19	0.040	0.034	0.001	16—left thalamus
**FES < HC**								
Right superficial amygdala	14 − 72 − 30	139	4.93	0.21	0.008	0.009	< 0.001	92—right cerebellum crus1

Whole-brain thresholded at *p* < 0.05, FDR corrected with a voxel extent of > 20; Only coordinates of highest peak in each cluster are reported.

### Correlations between altered functional connectivity and clinical variables

**Figure 2:**
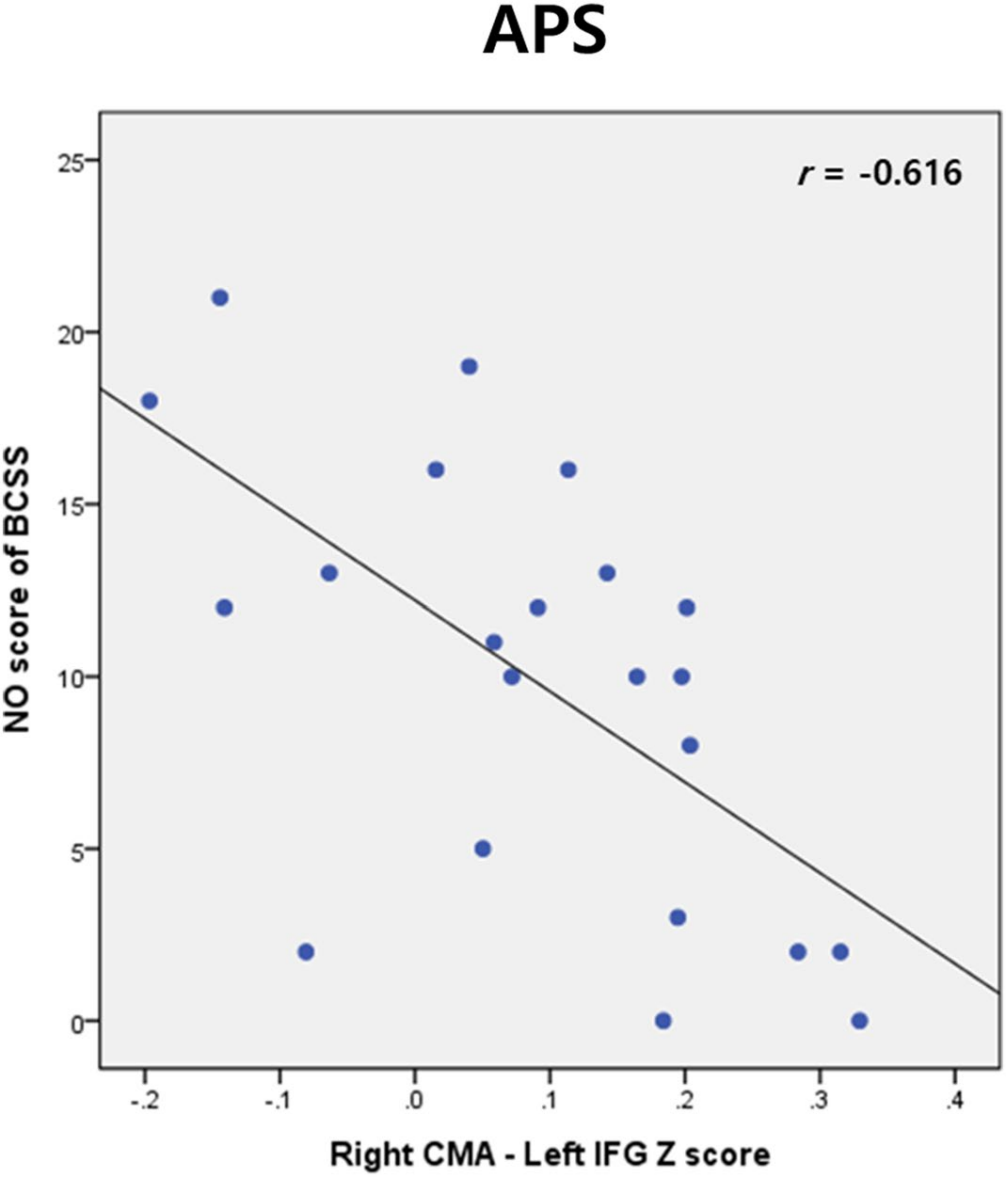
Associations between the right centromedial amygdala–left frontal pole cortex connectivity and negative-others scores of the Brief Core Schema Scale in the attenuated psychosis syndrome group.

In the APS group, the mean Z-value of the right CMA and the left FPC connectivity were negatively correlated with the BCSS negative-others score (*r* = − 0.58, *p* = 0.007) (Fig. 2). However, there were no significant correlations between altered FC and clinical variables in the FES group.

## Discussion

The AMY is a key component of a neural system specialized for rapid and automatic evaluation of stimuli that signal potential threat or danger in the environment. Typically, reduced AMY volume and activation to emotional stimuli have been reported in SZ. Using three subregions of the AMY as seed ROIs, we examined rsFC in individuals with APS, patients with FES, and HCs. Aberrant FC with the CMA was much wider and more prominent in the APS group, whereas disconnectivity with the LBA was only evident in SZ.

Individuals with APS showed increased connectivity between the right CMA and left FPC compared to HCs. An earlier study of individuals at high risk for SZ identified no significantly altered FC relative to HCs using the AMY as a single ROI^19^. However, if three subregions of the AMY had been used as seed ROIs, the results may have been different. The CMA plays a significant role in regulating attentional processing of cues during associative conditioning and generating emotional and associated physiological responses to threat or pain through projections to the brainstem, as well as the cortical and striatal regions^26,28^. Stimulation of the central nucleus of the AMY leads to fast, desynchronized cortical EEG activity, which is associated with increased attention and vigilance^29,43^. Projections from the central nucleus to the ventral tegmental area mediate stress-induced increases in dopamine metabolites in the prefrontal cortex^44^. The FPC occupying the most anterior part of the prefrontal cortex is functionally correlated with the default mode network^45^. Its role is specialized for disengaging cognitive control from the current task and re-distributing cognitive resources to other novel goals present in the environment^46^. At-risk and symptomatic individuals for psychosis show abnormalities in both structure^47^ and activation^48^ of the FPC. Considering the functions of the CMA and FPC, our finding suggests that individuals with APS are in an increased state of attention and vigilance to novel stimuli in the environment. This interpretation may be consistent with the hypothesis that psychosis is a state of aberrant assignment of salience to otherwise insignificant stimuli^49^.

Compared to HCs, individuals with APS were also found to have significant hyperconnectivity between the LBA and brain stem as well as the right inferior LOC. Increased connectivity of the LBA with the brain stem is supported by a previous study reporting that increased AMY connectivity with a brainstem was found in individuals at high risk for SZ^19^. The brainstem is home to a group of modulatory neurotransmitter pathways, such as those arising from the raphe nuclei (serotonergic), ventral tegmental area (dopaminergic), and locus coeruleus (noradrenergic), thereby controlling level-setting in sensory and arousal systems and emotional behaviors^50^. The LOC is involved in face perception^51^ or object recognition^52^. Therefore, our findings may be interpreted to indicate that individuals with APS who are exposed to negative facial stimuli are likely to exhibit physiological arousal. Unexpectedly, individuals with APS exhibited hypoconnectivity between the right CMA and right FPC compared to HCs. Several studies in which right FPC activity was measured via fMRI^53^ and EEG^54^ suggest that the right FPC plays a role in directed exploration. Hence, it may be that this decreased connectivity with right FPC could be associated with negative symptoms such as decreased exploration in individuals with APS.

In patients with FES, increased levels of connectivity were observed between the right SA and left OPC and between the left CMA and left thalamus compared to individuals with APS and HCs, respectively. The SA is implicated in olfactory and affective processes particularly related to facial emotions^55^. The occipital pole is the posterior-most part of the occipital lobe and is primarily responsible for visual processing. The traditional role of thalamus has been that of a passive relay station of information from sensory organs or subcortical structures to the cortex. However, its role has recently been extended to that of a critical hub region that could integrate diverse information being processed throughout the cerebral cortex as well as maintain the modular structure of cortical functional networks^56^. Increased connectivity between the AMY and thalamus in patients with FES is a relatively unique finding considering most of the previous studies on SZ reported decreased connectivity in amygdalo-^34^ or thalamo-cortical circuits^57^. Deficits in amygdala-orbitofrontal cortex coupling have been reported in both early course and chronic SZ^19^. Even though we did not observe altered amygdalo-cortical connectivity in FES group, our key finding is an aberrant connectivity between the AMY and sensory information processing regions, i.e., OPC and thalamus. This may suggest that individuals with FES are hypervigilant via sensory stimulation including facial perception. Moreover, it is in line with previous studies reporting strong connections between the AMY and auditory regions of the cortex and thalamus^58^ as well as disrupted pathways from limbic areas to thalamus in SZ^59^. Especially, it is interesting to note that strong connections between the AMY and auditory regions of the cortex and thalamus were shown in patients with SZ during the perception of aversive auditory stimuli mimicking the content of auditory verbal hallucinations^58^. The scores of P3 (hallucinatory behavior) in the PANSS were 1.82 ± 1.01 and 3.68 ± 1.77 for the APS and FES groups respectively. Therefore, it may be speculated that increased connectivity between the AMY and thalamus is associated with auditory verbal hallucinations in the FES group. It merits further investigation to see whether this aberrant connectivity between the AMY and thalamus is occurring in the APS group converted to psychosis.

Correlation analysis showed that the connectivity strength between the right CMA and left FPC was negatively related to the BCSS negative-others score in the APS group. This result seems counterintuitive considering our previous result from seed to voxel FC analysis, i.e. increased connectivity strength between the right CMA and left FPC in the APS group. A possible interpretation may be that although the strength of the connectivity between the CMA and FPC was stronger in individuals with APS than in HCs, greater connectivity is correlated with lower negative-others score within the APS group itself. Though clinical implication of inverse correlation within the APS group is ambiguous, it would be interesting to explore its relationship with conversion to psychosis. Alternatively, it may be that resting state brain activity does not reflect appropriately actual mental state, i.e., BCSS score and may be showing decompensated activity for negative schema. This study has several limitations. First, in the APS group, the sample size was small, and the sex ratio was male-biased. Second, because we used a cross-sectional design, there was no information about conversions to psychosis among individuals with APS. Therefore, the findings in APS should be interpreted cautiously. Third, some of the participants with FES were taking atypical antipsychotics, raising the possibility of a medication confound. Given the rapid and reversible effects of antipsychotic drugs on corticostriatal circuits^60^, future studies with drug-naive patients are required to eliminate medication effects.

Despite these caveats, this is the first trial to use three AMY subregions as seed ROIs to explore whole brain connectivity in individuals with APS. In conclusion, AMY-based voxel-wise analysis of FC with the rest of the brain demonstrated altered FC with the CMA and LBA in the APS group and the CMA and SA in the FES group compared to HCs. Especially for the CMA, increased connectivity with the FPC and thalamus was observed in the APS and FES groups, respectively. This suggests that the APS group may be hypervigilant to cognitive stimuli and FES group to sensory stimuli. Increased AMY connectivity with thalamus in the FES group may be associated with the development of auditory verbal hallucinations. Given the lack of specificity of many biological markers in psychiatric disorders, our findings suggest that FC analysis using a specific AMY subregion as a seed ROI could help uncover more specific neuroimaging biomarkers for psychosis.

## Methods

### Participants

Participants were help-seeking individuals with APS, patients with FES, and HCs. Diagnostic evaluation was performed by trained psychiatrists according to the Structured Clinical Interview for DSM-IV (SCID)^61,62^. For diagnosis and subtype classification of CHR states for psychosis, criteria of the DSM-5^63^ and Comprehensive Assessment of At-Risk Mental States (CAARMS)^64^ were employed. We recruited only the subjects with APS because it was most extensively studied syndrome within the psychosis continuum. The duration of illness (DI) for APS was calculated since the first significant attenuated psychotic symptoms appeared. First episode was defined as having experienced only a single episode of psychosis after the onset (individuals relapsing with another psychotic episode were excluded) and a DI of < 2 years. Patients or individuals with alcohol or drug use disorders within the past 6 months, intellectual disability (IQ ≤ 70), current or historical neurological disorders, a serious medical illness, pregnancy, and claustrophobia were excluded from the study. HCs were recruited via advertisements. The participants were interviewed using the screening module of the SCID-IV non-patient edition^62^ and were required to have no previous or current psychiatric disorders, neurological disorders, or significant medical conditions. Controls having a first-degree relative with a psychiatric disorder were also excluded. All participants were between 19 and 60 years of age and were assessed as right-handed using the Edinburgh Handedness Inventory^65^. They joined the study voluntarily and provided written informed consent. The study was approved by the Ethics Committee of Jeonbuk National University Hospital (approval number: CUH 2012-08-001). A statement confirming that all experiments were performed in accordance with relevant guidelines and regulations.

### Clinical assessment

The severity of symptoms was evaluated within a week of fMRI scanning using the Scale of Prodromal Symptoms^66,67^ (SOPS) or PANSS^40,41^. These scales were administered by trained psychiatrists. In addition, the BCSS^42^, a self-rating scale, was administered. The BCSS yields four subscale scores on negative-self, positive-self, negative-others, and positive-others schemas. A higher score indicates a greater endorsement of a schema. This scale was chosen on the basis of the close association between negative schemas and positive symptoms^39^.

### Image acquisition and preprocessing

Resting-state functional and structural MRI data were obtained at the Jeonbuk National University Hospital on a 3 T Verio scanner (Siemens Magnetom Verio, Erlangen, Germany) using a 12-channel standard quadrature head coil. The three-dimensional T1-weighted image was acquired using magnetization-prepared rapid gradient echo (repetition time [TR]: 1900 ms, echo time [TE]: 2.5 ms; flip angle: 9°; field of view [FOV]: 250 mm; image matrix: 256 × 246 mm; voxel size = 1.0 × 1.0 × 1.0 mm^3^; 176 slices). For each participant, we collected a 5-min resting-state scan consisting of 150 contiguous echo-planar imaging functional images (TR: 2000 ms; TE: 30 ms; flip angle: 90°; FOV: 220 mm; image matrix: 64 × 64 mm; voxel size = 1.0 × 1.0 × 1.0 mm^3^; 26 slices). During resting-state image acquisition, the participants were asked to relax with their eyes closed and not to sleep. MRI data processing was conducted using Statistical Parametric Mapping software package, version 12 (SPM12; www.fil.ion.ucl.ac.uk/spm; Wellcome Department of Cognitive Neurology, London, UK) implemented in MATLAB. The first three volumes were discarded to adjust for magnetization equilibrium effects. Functional images were slice-time corrected, realigned to correct for head motion artifacts, and co-registered with each participant’s structural image. Then, the co-registered functional data were transformed into a standard anatomical space based on the parameters obtained by spatially normalizing each T1 image to the Montreal Neurological Institute (MNI) template. Normalized images were smoothed with an 8-mm full-width at half-maximum isotropic Gaussian kernel. The criteria for excessive head motion were translation > 2 mm or rotation > 2° in any direction and frame displacement (FD) values > 0.5 mm. Participants for whom more than 10% of volumes showed excessive head motion were excluded from the analysis^68^. FD values were computed using the FSL toolbox (https://www.fmrib.ox.ac.uk/fsl/index.html). Head motion was measured in six dimensions and the component correction (CompCor)^69^ noise components were used as nuisance variables. The CompCor built into the CONN toolbox (V 14f., https://www.nitrc.org/projects/conn) was used to increase the accuracy of grey matter (GM) signals by removing physiological noises such as heart rate and breathing signals, followed by the removal of the main components from the white matter (WM) and cerebrospinal fluid (CSF) signals. Afterward, the linear trend was removed through the time course, and the band-pass filter (0.008 < f < 0.09 Hz) was applied.

### Functional connectivity analysis

The SPM Anatomy toolbox^70^ (V 2.2c) was used to parcellate the AMY into three ROIs, i.e., CMA, LBA, and SA^71^. After applying predefined ROIs to the image data of each subject, we used MANGO (https://ric.uthscsa.edu/mango/mango.html) to check whether they matched well. For each ROI, the BOLD time series of the voxels within the ROI were averaged to generate the reference time series for the ROI. FC analysis was performed using correlation analysis between the seed amygdala ROI and other voxels of the entire brain in a voxel-wise manner using the CONN toolbox. Group comparison was performed using a one-way analysis of variance (ANOVA) with sex and education as covariates. Direct group comparisons were further evaluated using post-hoc *t-*tests. In both analyses, we applied the voxel-level height threshold of uncorrected p < 0.001 and the cluster-level extent threshold of p < 0.05, which was corrected for multiple comparisons using the false discovery rate (FDR). To control the probability of a type I error, we further performed 10,000 permutation tests implemented in the CONN toolbox.

### Statistical analysis

Demographic and clinical data were compared among the three groups using ANOVA, *t*-test, or Chi-square test depending on the variables. For the correlation analyses, the Region of Interest Extraction Tool (https://software.incf.org/software/rex) in the CONN toolbox was used to extract Fisher's Z transformed signal intensity values for brain regions with significant group differences. Relationships between the PANSS, BCSS, or SOPS score and z-scores were explored using sex and education as covariates with SPSS 20.0. Multiple comparison corrections were performed using the Bonferroni correction.
